# Timing of periarticular injection has no effect on postoperative pain and functional recovery in simultaneous bilateral total knee arthroplasty: a prospective randomized, double-blinded trial

**DOI:** 10.1186/s12891-019-2526-z

**Published:** 2019-04-11

**Authors:** Artit Laoruengthana, Atthakorn Jarusriwanna, Piti Rattanaprichavej, Supachok Rasamimongkol, Panapol Varakornpipat, Krit Pongpirul

**Affiliations:** 1 Department of Orthopaedics, Faculty of Medicine, Naresuan University, 99 Moo 9, Phitsanulok-Nakhon Sawan Road, Tha Pho, Mueang Phitsanulok, Phitsanulok, 65000 Thailand; 2 Department of Preventive and Social Medicine, Faculty of Medicine, Chulalongkorn University, 1873 Rama IV Road, Pathum Wan, Pathum Wan, Bangkok, 10330 Thailand; 3 Department of International Health, Johns Hopkins Bloomberg School of Public Health, 615 N. Wolfe Street, Baltimore, MD 21205 USA

**Keywords:** Total knee arthroplasty, Simultaneous bilateral total knee arthroplasty, Periarticular multimodal drug injection, Preemptive analgesia, Knee osteoarthritis

## Abstract

**Background:**

Given no consensus on optimal timeframe of periarticular multimodal drug injection (PMDI) in knee osteoarthritis patients undergoing total knee arthroplasty (TKA), this study was aimed to compare the postoperative pain and the functional recovery in patients who underwent simultaneous bilateral TKA (SBTKA) and received PMDI at the different intraoperative time points.

**Methods:**

This prospective, randomized, double-blinded controlled trial study included 48 patients who underwent SBTKA and received PMDI mixture, either before prosthetic implantation (late PMDI), or just after knee arthrotomy (early PMDI). Each subject’s knees were randomly selected to different PMDI administration time points. The outcome parameters were postoperative pain assessed by using a visual analog scale (VAS), the maximal angle of knee flexion, and quadriceps function from day 1 to 6 weeks after surgery.

**Results:**

Late PMDI revealed slightly higher VAS at 6 and 12 h after the operation than early PMDI administration. Afterward, the VAS tended to be lower in the late than early PMDI administration until the end of the study, but without statistical significance. The time difference between early and late PMDI had no effect on postoperative VAS, while older age resulted in significantly less pain. No statistical differences between the two groups in all other outcome parameters were observed.

**Conclusions:**

Postoperative pain reduction and functional recovery of SBTKA with early and late PMDI administration were not significantly different. The time interval of PMDI between knees did not confound the comparison of postoperative pain and functional recovery in SBTKA.

**Trial registration:**

The protocol of this study was retrospectively registered in the Thai Clinical Trials Registry database No.TCTR20170617001 on 16 June 2017.

## Background

Total knee arthroplasty (TKA) is one of the most frequently performed surgical procedures by orthopedic surgeons, with effective treatment and a high success rate in patients suffering from knee osteoarthritis [1, 2]. However, previous literature reported the incidence of moderate-to-severe pain after TKA at 30 days following surgery was about 50% and approximately 20% of the patients were dissatisfied with the outcomes following the operation [3, 4]. Thus, postoperative pain has become a major concern for the patients, as well as surgeons. There are many analgesic modalities including epidural analgesia, intrathecal and intravenous opioids, and peripheral nerve block proposed for improvement of postoperative pain management [5–8].

Exaggerated pain response following TKA is caused by both peripheral and central sensitization, which may persist up to 3 months after surgery [9]. Prevention of hyperexcitability stage of peripheral and central nociceptors is a principle of preemptive analgesia that could consequently reduce amplification of postoperative pain [3, 5, 10]. Strategies that provide an adequate blockade to prevent neural hypersensitization and extend the effect during the inflammatory response in the early postoperative period are necessary [11, 12], and thus the multimodal pain management that consists of preemptive, perioperative, and postoperative analgesia is widely accepted for its efficacy in postoperative pain control [13].

Currently, periarticular multimodal drug injection (PMDI) is trendiness because of its simplicity, effectiveness, and limited adverse effects [14, 15]. The periarticular injection has been reported of providing better pain relief and diminishing of opioid-related adverse effect in the first 24 h after simultaneous bilateral total knee arthroplasty (SBTKA) when compared to epidural analgesia [16]. By improving the efficacy of the PMDI, the proper location of periarticular injection can reduce postoperative pain and improve quadriceps function [17]. The ingredients of the mixture could also influence on pain scores, opioid consumption, and range of motion following TKA [18]. Nevertheless, there is neither consensus regarding the appropriate time of periarticular injection during the knee replacement procedure nor the effect of the interval between PMDI in postoperative pain and functional recovery after SBTKA. The purpose of this study was to investigate and compare the postoperative pain and functional recovery (range of knee motion and quadriceps function) and assess the confounding effect of the interval between PMDI in patients who underwent SBTKA and received the PMDI in the different intraoperative timeframe.

## Methods

This study was a prospective randomized controlled, double-blinded trial of patients with the diagnosis of bilateral primary knee osteoarthritis and scheduled for SBTKA. The study protocol was approved by the Institutional Review Board of Naresuan University. The written informed consents were obtained before the enrollment. Patients who had any previous knee surgery, knee infection, inflammatory joint disease, history of the thromboembolic event, or a neuromuscular disorder, were excluded from the study.

**Fig. 1:**
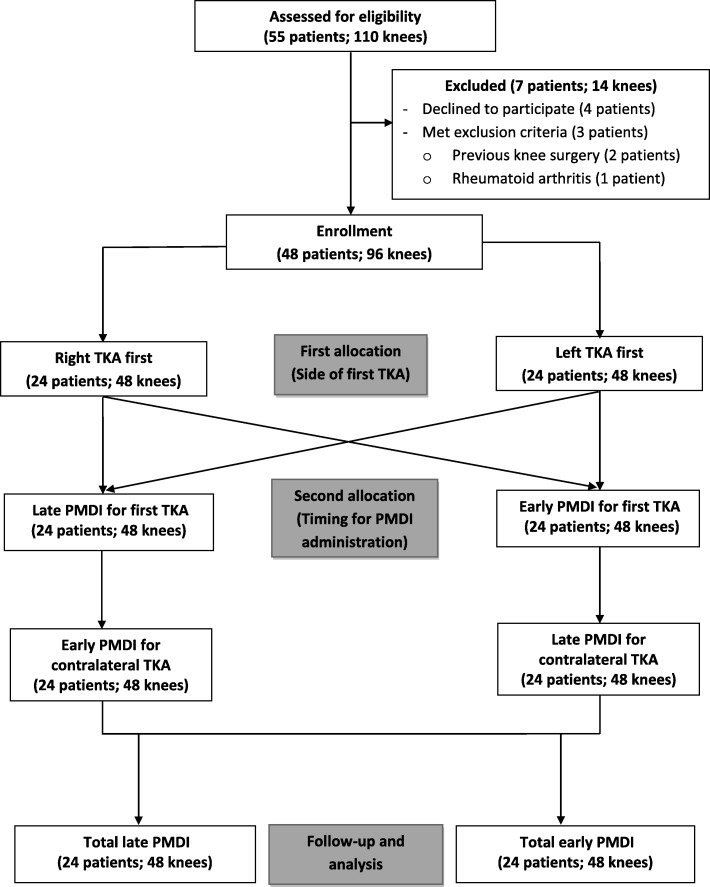
Flow diagram of patients in this study

Fifty-five patients who were scheduled for SBTKA were assessed for the study. Of 55 patients, seven patients were excluded (four patients declined to participate in the study, two patients had previous knee surgery, and one patient had an underlying disease of rheumatoid arthritis). The remaining 48 patients had enrolled and completed the study. All surgical procedures were performed by a single surgeon under spinal anesthesia with 2.8–3.6 mL of bupivacaine (0.5% Marcaine, AstraZeneca, Sweden). The cemented fixed-bearing, posterior stabilized, knee prostheses were implanted via a standard medial parapatellar approach in all cases. The PMDI mixture consisting of 100 mg bupivacaine, 30 mg Ketorolac (Ketolac 1 mL, SiuGuan, Taiwan), 5 mg morphine sulfate, and 300 μg adrenaline (1:1000, 0.3 mL) were diluted with a sterile normal saline solution to a total volume of 150 mL. The surgery was randomized using a sealed envelope to start on one side and the second randomization determined two different timings of PMDI that would be administered in the first operated knee. A group of “late PMDI” was the knees which received 75 mL of PMDI mixture prior to the prosthetic implantation as common practice of surgeons, whereas a group of “early PMDI” received 75 mL of the mixture immediately after knee arthrotomy (prior to osteophyte removal, medial collateral ligament release, synovectomy, initial bone cut, and meniscectomy). Therefore, the contralateral knee would receive the PMDI in the opposite timing. The time lag between PMDI administration was recorded and defined as the interval between PMDI. The flow diagram of patients in the study was shown in Fig. 1. Identical postoperative care was utilized in all patients. For the first 48 h after surgery, prophylactic antibiotics and intravenous patient-controlled analgesia (PCA) opioid were applied. After 48 h postoperatively, the antibiotics, morphine PCA, all catheters, including a surgical drain, were discontinued. Additional 2 mg of morphine and 30 mg of ketorolac were given intravenously every 8 and 12 h, respectively, until 72 h after the surgery. Identical rehabilitation protocol was administered for all patients including a continuous passive motion (CPM) device applied on the day after surgery, and walking with a walker was allowed on day 2 postoperatively. All patients received low molecular weight heparin for the first 48 h, combined with oral warfarin for 10 days.

All patients were assessed and evaluated by two independent investigators who were blinded to the randomized process and the surgical procedures. Postoperative pain assessment using the 10-cm visual analog scale (VAS) was recorded during rest at 6, 12, 24, 48, 72, 96 h, and at 2 and 6 weeks after surgery. The maximal angle of knee flexion reached by the CPM device was noted every day during hospitalization and the active knee flexion angle was measured with a goniometer at 2 and 6 weeks postoperatively. The postoperative quadriceps function was determined by the degree of active straight leg raising (SLR) and extension lag (EL). The operative time, drain output, and the patient’s preferred side at the last admission day before discharge from the hospital, and at 2 and 6 weeks after surgery were also recorded.

### Statistical analysis

All measured demographics and clinical outcomes were summarized with descriptive statistics including mean and standard deviation. Normality of data was checked with the Kolmogorov-Smirnov test. We compared the outcomes between groups by using the student’s t-test for normally distributed data, and using the Wilcoxon matched-pairs signed rank test if the data were not normally distributed. Generalized estimating equations (GEE), adjusted for age, body mass index (BMI), American Society of Anesthesiologists (ASA) physical status classification, and interval between PMDI, was used for comparing the change of pain scores, knee flexion angle, SLR, and EL between the early and late PMDI along the study period (prior to the operation and at 6, 12, 24, 48, 72, 96 h, and at 2 and 6 weeks after surgery). Statistical significance was defined as *p*-value < 0.05. A sample size of 48 knees for each group was anticipated to detect a difference in VAS between early and late PMDI, with a standard deviation of 1.5 [17], 90% power, and a significance level alpha of 0.05. Collecting repeated measurements of the outcome variables with GEE analysis accounted for within-person change across time, resulted in increased statistical power for detecting changes while reducing the costs of conducting a study. Stata/MP 15.0 software (StataCorp LP, College Station, TX, USA) was used for the analyses.

## Results

**Fig. 2:**
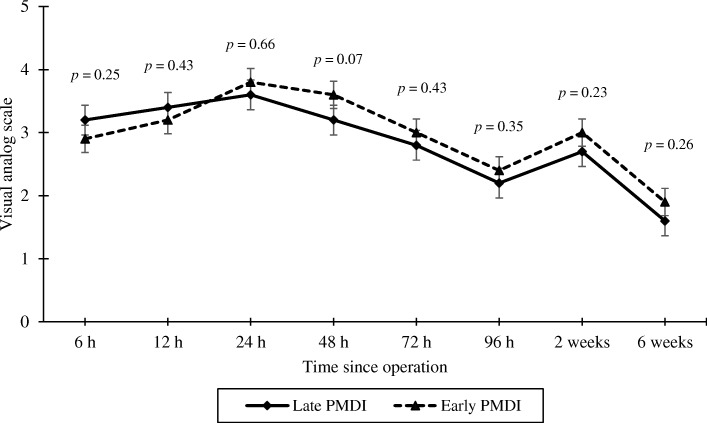
Postoperative pain assessment by visual analogue scale (VAS)

A total of 48 patients with bilateral knee osteoarthritis undergoing SBTKA were included in the study. Patients’ demographics and baseline characteristics were provided in Table 1. Mean age of the patients was 62.9 years, with predominant female gender (93.8%). No statistical differences between groups in the preoperative VAS, the degree of active knee flexion, the operative time, and the drain output were observed. The interval between PMDI averaged 64.7 ± 39.7 min. The late PMDI administration revealed higher VAS at rest in the first 12 h after surgery. However, the VAS tended to be lower than the early PMDI afterward, until the end of the study without significance (*p* > 0.05 at any time point) as shown in Fig. 2. There was no statistical difference in other parameters as detailed in Table 2. These findings were confirmed by a GEE analysis, the knees with early PMDI had 0.06 (95% confidence interval (CI) −0.41 to 0.53; *p* = 0.80) higher VAS than the late PMDI. Interval between PMDI, BMI, and ASA physical status classification had −0.003 (95% CI −0.01 to 0.003; *p* = 0.29), 0.00 (95% CI −0.08 to 0.08; *p* = 0.98), and −0.45 (95% CI −1.02 to 0.12; *p* = 0.13) changing of VAS scores, respectively, while each additional year of patient age significantly reduced VAS score by 0.05 points (95% CI −0.10 to −0.01; *p* = 0.01). However, all these factors had no effect on the change of other outcomes throughout the study period.

**Table 1:** Patients’ demographic data

Demographic data	Values	*P*-value
Number of patients	48	
Age^a^ (years)	62.9 ± 5.8	
Gender (male: female)	3: 45	
BMI^a^ (kg/m^2^)	25.9 ± 3.3	
Length of stay^a^ (days)	7.3 ± 1.4	
The side of the first operated knee (right: left)	24: 24	
The timing of PMDI for the first knee (late: early)	24: 24	
Preoperative VAS^a^		
Late PMDI	6.8 ± 2.5	
Early PMDI	6.7 ± 1.9	0.77
Preoperative degrees of active knee flexion^a^		
Late PMDI	114.6 ± 19.1	
Early PMDI	114.9 ± 18.3	0.90

^a^Data are presented as mean and standard deviation (SD)

Late PMDI, the knees injected with the PMDI before prosthetic implantation as common practice of surgeons

Early PMDI, the knees injected with the PMDI after arthrotomy to prevent neural hypersensitization caused by further soft tissue and bone work during TKA

**Table 2:** The perioperative and postoperative characteristics between the knees with late and early PMDI administration

Parameters	Late PMDI	Early PMDI	Z	*P*-value
Operative duration (min)	65.9 (62.9–68.9)	66.8 (63.3–70.3)	−0.73	0.47
Drain output (mL)				
24 h	182.7 (140.3–225.1)	156.4 (112.9–199.9)	−1.49	0.14
48 h	137.7 (93.0–182.4)	136.1 (93.0–179.2)	−0.38	0.71
VAS at rest				
6 h	3.2 (1.9–4.5)	2.9 (1.9–3.9)	−1.16	0.25
12 h	3.4 (2.3–4.5)	3.2 (2.2–4.2)	−0.79	0.43
24 h	3.6 (2.7–4.5)	3.8 (2.8–4.8)	−0.44	0.66
48 h	3.2 (2.3–4.1)	3.6 (2.8–4.4)	−1.83	0.07
72 h	2.8 (2.0–3.6)	3.0 (2.2–3.8)	−0.79	0.43
96 h	2.2 (1.5–2.9)	2.4 (1.9–2.9)	−0.93	0.35
2 weeks	2.7 (1.8–3.6)	3.0 (2.2–3.8)	−1.20	0.23
6 weeks	1.6 (0.9–2.3)	1.9 (1.1–2.7)	−1.12	0.26
Degrees of knee flexion using CPM				
24 h	53.8 (45.7–61.9)	53.2 (45.1–110.0)	−0.55	0.59
48 h	74.2 (66.1–82.3)	72.8 (63.6–82.0)	−1.22	0.22
72 h	83.3 (78.2–88.4)	81.7 (76.0–87.4)	−1.46	0.15
96 h	87.2 (80.4–94.0)	87.2 (80.4–94.0)	−0.19	0.85
Degrees of active knee flexion				
2 weeks	105.8 (99.3–112.3)	104.3 (96.0–112.6)	−0.21	0.84
6 weeks	115.7 (111.0–120.4)	114.4 (109.4–119.4)	−1.53	0.13
Degrees of straight leg raising (SLR)				
48 h	30.8 (16.0–45.6)	31.6 (16.6–46.6)	−0.54	0.59
72 h	32.2 (18.6–45.8)	33.6 (20.1–47.1)	−0.32	0.75
96 h	34.0 (20.1–47.9)	36.2 (22.3–50.1)	−0.57	0.57
2 weeks	42.2 (27.3–57.1)	43.6 (27.4–59.8)	−0.03	0.97
6 weeks	55.0 (38.4–71.6)	53.2 (35.5–70.9)	−0.64	0.52
Degrees of extension lag (EL)				
48 h	21.3 (17.8–24.8)	20.5 (16.3–24.7)	−0.57	0.57
72 h	19.6 (16.2–23.0)	19.3 (15.4–23.2)	−0.46	0.65
96 h	15.3 (11.7–18.9)	14.7 (11.2–18.2)	−0.73	0.47
2 weeks	14.9 (12.2–17.6)	15.4 (12.3–18.5)	−0.29	0.77
6 weeks	7.1 (4.7–9.5)	7.0 (4.8–9.2)	−0.36	0.72

All parameters are presented as mean and 95% confidence interval (CI)

Late PMDI, the knees injected with the PMDI before prosthetic implantation as common practice of surgeons

Early PMDI, the knees injected with the PMDI after arthrotomy to prevent neural hypersensitization caused by further soft tissue and bone work during TKA

On the last admission day, 21 patients (43.8%) preferred the knee with early PMDI administration, while 16 patients (33.3%) preferred the side with late PMDI, and 11 patients (22.9%) could not differentiate between both knees. At 2 weeks postoperatively, 14 (29.2%) and 18 (37.5%) patients preferred the knee injected immediately after the arthrotomy, and just prior to the implantation, respectively, and 16 patients (33.3%) felt similarly. At 6 weeks after surgery, most of the patients (26 patients, 54.2%) could not feel a difference between both knees. Four patients experienced minor complications. In both groups, superficial wound infection was suspected and managed by oral antibiotics prophylaxis. One deep vein thrombosis (DVT) was questionable in each group which further study by venous ultrasonography confirmed no clotting. These clinical findings were improved later without specific treatment.

## Discussion

Postoperative pain management is one of the essential elements in improving the patient’s satisfaction, rehabilitation, and functional recovery following the TKA, especially in SBTKA. The PMDI is commonly administered before implantation of the prosthesis or before wound closure by most surgeons [19, 20]. However, the appropriate time of multidrug mixture injection remains inconclusive [13]. Based on the preemptive analgesia, administration of the PMDI as early as possible may pretreat postoperative pain and minimize the nervous system response. Motififard et al. [21] conducted a study to demonstrate the effectiveness of preemptive PMDI technique in patients undergoing TKA. Their study reported the PMDI, which was given 15 min prior to the incision, would result in better post-TKA pain relief, postoperative rehabilitation, and reduction of opioid consumption at 48 h after surgery with better function during postoperative 6 weeks. However, their control group received a periarticular injection with 300 μg of epinephrine (1:1000) that diluted with 0.9% sodium chloride solution. Thus, the superior effect of the preemptive PMDI in their study might be attributed by the effect of bupivacaine, morphine, and ketorolac in the cocktail mixture administered for the study group.

According to our study, we injected a similar multidrug mixture into the identical location of both knees under direct vision, but at a different timeframe. Therefore, we could directly compare the efficacy between early and late administration of PMDI. We found that the knees receiving the PMDI immediately after arthrotomy had slightly lower drain output at the day after surgery, and lower pain scores at postoperative 6 and 12 h when compared to the other group without statistical significance. The PMDI that was delivered just before implantation seemed to reduce pain better than early administration of PMDI at 24 h and afterward. However, there was no statistical significance along the study period.

In addition, the previous study showed that the first operated knee would have higher pain scores significantly with less patient satisfaction compared to the latter side in SBTKA [22]. In that study, a single surgeon always performed the first operation on the right knee. They reported 73.9% of patients were satisfied with the left knee more than another side. Although, there were various studies assessing the outcomes of periarticular injection between knees in SBTKA with different techniques [17, 23, 24], there was a paucity of information in the confounding effect of the time lag between the injections. In this study, we assessed the confounding effect of the interval between PMDI and noticed that this factor did not confound the comparison of postoperative VAS, the degree of knee flexion, SLR, and EL between knees within the same patient at any point of time.

Currently, multimodal pain management is widely accepted as a principle of contemporary pain management [5, 13]. Several classes of oral and intravenous medications have been applied to maximize pain relief and opioid-sparing effect. The use of nonsteroidal anti-inflammatory drugs (NSAIDs) and pregabalin as the preemptive analgesia has revealed better postoperative pain control, reduction of opioid consumption, and greater functional recovery [25–27]. Munteanu et al. [26] compared the cumulative morphine consumption during 48 h after TKA between patients who received a 120 mg of etoricoxib at 1 h before surgery, at the end of surgery, and a placebo group. The result showed preemptive administration of etoricoxib is superior to either postoperative administration or placebo in terms of morphine-sparing effect. Lee et al. [27] reported the patients who received a preoperative single dose of 150 mg pregabalin had significantly lower pain scores at 6 and 12 h after surgery and lower fentanyl consumption during the first 48 h postoperatively when compared to the control group.

This study has some limitations. First, the participants were predominantly female gender, which might be a factor affecting the postoperative pain and function [28]. In our findings, age was the only factor that affected the postoperative VAS scores after adjusting for BMI, ASA physical status classification, the timing of PMDI, and the interval between PMDI. However, the recent systemic review could not identify gender and age as significant predictors of poor outcome of pain and function after TKA [29]. Second, the preemptive analgesia may not be as convenient as “prior to the incision”, but it can administer prior to the surgery, during the surgery, or immediately after the surgery whenever it provides an adequate blockade to prevent neural hypersensitization [11, 12]. In our study, we could not prevent neural sensitization initiated from the skin incision and arthrotomy because we aimed to avoid a serious confounding effect of blinded periarticular injection. Finally, using an injection mixture with different composition and concentration might affect the outcomes.

## Conclusions

The early administration of the PMDI is able to slightly reduce pain in the first 12 h after surgery without statistical significance. In the same way, late administration of the PMDI is not able to significantly extend the period of pain relief. Therefore, the results suggest that the timing of the PMDI has no effect on pain relief and functional recovery following TKA. Additionally, the interval of PMDI between knees is not a confounding factor for comparison of the postoperative pain score and functional recovery following SBTKA.

## Abbreviations

ASA: American Society of Anesthesiologists
BMI: Body mass index
CI: Confidence interval
CPM: Continuous passive motion
DVT: Deep vein thrombosis
EL: Extension lag
GEE: Generalized estimating equations
NSAIDs: Nonsteroidal anti-inflammatory drugs
PCA: Patient-controlled analgesia
PMDI: Periarticular multimodal drug injection
SBTKA: Simultaneous bilateral total knee arthroplasty
SLR: Straight leg raising
TKA: Total knee arthroplasty
VAS: Visual analog scale
